# Incidence and risk factors of asymptomatic bacteriuria in patients with type 2 diabetes mellitus: a meta-analysis

**DOI:** 10.1007/s12020-023-03469-6

**Published:** 2023-08-21

**Authors:** Mengqiao Dai, Shan Hua, Jiechao Yang, Dandan Geng, Weina Li, Shuqin Hu, Hu Chen, Xiaoqin Liao

**Affiliations:** 1 Shanghai University of Traditional Chinese Medicine, School of Nursing, Shanghai, 201203 China; 2 Department of Urology, Shanghai General Hospital, Shanghai Jiao Tong University School of Medicine, Shanghai, China

**Keywords:** Type 2 diabetes, Asymptomatic bacteriuria, Risk factor, Incidence, Meta-analysis

## Abstract

**Background:**

The prevalence of type 2 diabetes mellitus (T2DM) is increasing each year and has become one of the most prominent health concerns worldwide. Patients with T2DM are prone to infectious diseases, and urinary tract infections are also widespread. Despite a comprehensive understanding of urinary tract infection (UTI), there is a lack of research regarding primary prevention strategies for asymptomatic bacteriuria (ASB).

**Objective:**

To clarify the incidence and risk factors of asymptomatic urinary tract infection in patients with T2DM by meta-analysis to provide evidence for preventing UTI. Help patients, their families, and caregivers to identify the risk factors of patients in time and intervene to reduce the incidence of ASB in patients with T2DM. Fill in the gaps in existing research.

**Study design:**

Meta-analyses were conducted in line with PRISMA guidelines.

**Methods:**

Eleven databases were systematically searched for articles about ASB in T2DM, and the retrieval time was selected from the establishment of the database to February 5, 2023. Literature screening, quality evaluation, and meta-analysis were independently performed by two researchers according to the inclusion and exclusion criteria, and a meta-analysis was performed using Stata 17.0.

**Results:**

Fourteen articles were included, including cohort and case–control studies. A meta-analysis of 4044 patients with T2DM was included. The incidence of ASB in patients with T2DM was 23.7%(95% CI (0.183, 0.291); *P* < 0.001). After controlling for confounding variables, the following risk factors were associated with ASB in patients with T2DM: age (WMD = 3.18, 95% CI (1.91, 4.45), *I*^2^ = 75.5%, P < 0.001), female sex (OR = 1.07, 95% CI(1.02, 1.12), *I*^2^ = 79.3%, *P* = 0.002), duration of type 2 diabetes (WMD = 2.54, 95% CI (1.53, 5.43), *I*^2^ = 80.7%, *P* < 0.001), HbA1c (WMD = 0.63, 95% CI (0.43, 0.84), *I*^2^ = 62.6,%. *P* < 0.001), hypertension (OR = 1.59, 95% CI (1.24, 2.04), *I*^2^ = 0%, <0.001), hyperlipidemia (OR = 1.66, 95% CI (1.27, 2.18), *I*^2^ = 0%, *P* < 0.001), Neuropathy (OR = 1.81, 95% CI (1.38, 2.37), *I*^2^ = 0%, *P* < 0.001), proteinuria (OR = 3.00, 95% CI (1.82, 4.95), *I*^2^ = 62.7%, *P* < 0.001).

**Conclusion:**

The overall prevalence of ASB in T2DM is 23.7%. Age, female sex, course of T2DM, HbA1C, hypertension, hyperlipidemia, neuropathy, and proteinuria were identified as related risk factors for ASB in T2DM. These findings can provide a robust theoretical basis for preventing and managing ASB in T2DM.

## Introduction

Type 2 diabetes mellitus (T2DM) is a metabolic disease characterized by hyperglycemia caused by insulin resistance or insufficient insulin secretion. Clinical features include polydipsia, polyuria, and weight loss [1, 2]. Long-term hyperglycemia will lead to organ damage and dysfunction, including vision loss, renal failure, cardiovascular diseases, sexual dysfunction, gastrointestinal and urinary tract infections, and so on [3]. According to a survey conducted by the International Diabetes Federation in 2021, there are ~537 million diabetes patients worldwide, an increase of 16% compared to 2019 [4]. Diabetes reduces the immune function of individual patients and is prone to vascular diseases, causing microcirculation blood flow disorders, leading to tissue hypoxia, and a decrease in antibody distribution leads to infection. High blood sugar levels are also conducive to the survival of pathogens such as bacteria and fungi [5, 6]. Therefore, patients with diabetes are more prone to infections, including urinary tract infections, which are often caused by gram-negative bacilli (G-), Enterobacter, and Klebsiella [7]. Urinary tract infections (UTI) include symptomatic bacteriuria and asymptomatic bacteriuria, which leads to a decline in patients’ quality of life and an increase in social and psychological burden [8]. Asymptomatic bacteriuria (ASB) is also a disease that cannot be ignored. Studies have shown that the incidence of ASB in patients with diabetes is approximately three times that in normal people [9]. The Infectious Diseases Society of America believes that diabetic patients do not need to be screened or treated for asymptomatic bacteriuria. Screening or treatment will not improve the symptoms or outcome. Contrary, intervention with antibiotics will make the intestinal flora of diabetic patients disorder and lead to weakness. The application of antibiotics may lead to antibiotic resistance and clostridioides difficile infection [10]. However, ASB will develop into UTI, which will damage the patient’s urinary system. It is necessary and meaningful to carry out primary prevention for diabetic patients without screening or treating asymptomatic bacteriuria. Through meta-analysis, the risk factors can be obtained, so as to achieve accurate prevention and intervention and reduce the incidence of ASB in T2DM.

ASB patients have no symptoms or signs of urinary tract infection but are positive after repeated urine bacterial cultures; in these patients, the number of colonies after two consecutive cultures reaches 105/ml [11]. Currently, healthcare professionals pay more attention to symptomatic bacteriuria but less to the study of ASB. Long-term ASB can damage the renal function of patients with diabetes, and approximately half of ASB patients will develop symptomatic bacteriuria [12]. Strengthening the primary prevention of ASB in patients with diabetes can reduce the incidence of ASB, thereby reducing the possibility of symptomatic bacteriuria and improving the quality of life of patients with diabetes [13]. This study systematically searched for related cohort and case–control studies on ASB in T2DM patients and conducted a meta-analysis to screen for related risk factors. This study aims to generate evidence to help patients, their families, and medical workers prevent asymptomatic bacteriuria and to increase the evidence for identifying patients with diabetes with a high risk of ASB. The calculation of the prevalence rate fills the gap in the epidemiology of ASB in T2DM, provides a reference for clinical medical staff, offers new research ideas for researchers, and improves the understanding of ASB.

## Methods

### Literature search strategy

Eleven databases, including PubMed, Web of Science, Cochrane, Embase, Ovid, Scoups, Chinese full-text journal database, VIP database, Wan Fang database, The National Medical Journal of China, and Sinomed database, were queried, and Endnote X9 software was used to screen the standard literature. Stata 17.0 software was used to perform a meta-analysis of the included literature. The retrieval period was from the establishment of the database to February 5, 2023. The retrieval strategy adopted a combination of subject and free words. For data retrieval from Chinese databases, we used the keywords “asymptomatic bacteriuria,” “occult bacteriuria,” “bacteriuria,” “pyuria,” “Leukocyturia,” “asymptomatic urinary tract infection,” “occult urinary tract infection,” “asymptomatic bladder infection,” “occult bladder infection,” “asymptomatic ureteral infection,” “occult ureteral infection” and “type 2 diabetes mellitus.” The retrieval strategy for the English database is shown in the Appendix in detail. The reference lists of relevant articles were screened and checked to identify eligible studies.

### Inclusion criteria

Patients: (1) Type 2 diabetes mellitus with asymptomatic bacteriuria diagnosed definitively; (2) age ≥18 years; and (1) Case-control study or cohort study. (2) The language of the literature is Chinese or English.

### Exclusion criteria

(1) Abnormal urinary system structure; (2) Document types such as conference reports, case reports, and reviews; (3) inability to obtain complete literature, repeated publications, or incorrect statistical methods; (4) Newcastle Ottawa Scale Score (NOS) < 6.

### Data extraction and quality assessment

After importing articles into Endnote X9, the titles and abstracts of the articles were independently read and screened by two researchers (MengQiao Dai and Shan Hua) who had systematically studied evidence-based courses. The remaining documents were independently read and screened according to the exclusion criteria. The two researchers independently extracted data from the literature that met the inclusion criteria, including author information, publication year, risk factors, literature type, sample size, age, prevalence rate, and related risk factors. While selecting articles and extracting information, if two researchers disagreed, a third researcher with the same qualification was consulted to reach a consensus to ensure the quality of the included articles.

Two researchers independently evaluated the quality of the articles and scored them using the NOS scale. The NOS score includes three large blocks, with eight items to evaluate case-control or cohort studies, including the selection, comparability, exposure, evaluation, and results of the research population. The total score was 9, and articles with a score of less than 6 were excluded. If two researchers disagreed on the quality evaluation of an article, a third researcher with the same qualification was consulted to reach a consensus.

### Statistical analyses

Stata 17.0 (StataCorp LLC, College Station, Texas, USA) was used to analyze the data. The odds ratio (OR) was used as the effect index for counting data, the weighted mean difference (WMD) was used as the effect index for measuring data. The interval estimation was expressed as a 95% confidence interval. If the heterogeneity test showed *P* ≥ 0.1 and *I*^2^ ≤ 50% suggested that the study was homogeneous, the fixed effect model was adopted. Studies were considered heterogeneous if the heterogeneity was *P* < 0.1 and *I*^2^ > 50%. The random effects model was used to determine the source of heterogeneity through sensitivity analysis or subgroup analysis. If the heterogeneity was still large, a random-effect model combined with a descriptive analysis was used to express the results. According to the Cochrane website recommendations, a funnel chart was used for publication bias analysis when a single risk factor was present in more than ten articles.

## Result

### Results of literature retrieval

**Fig. 1:**
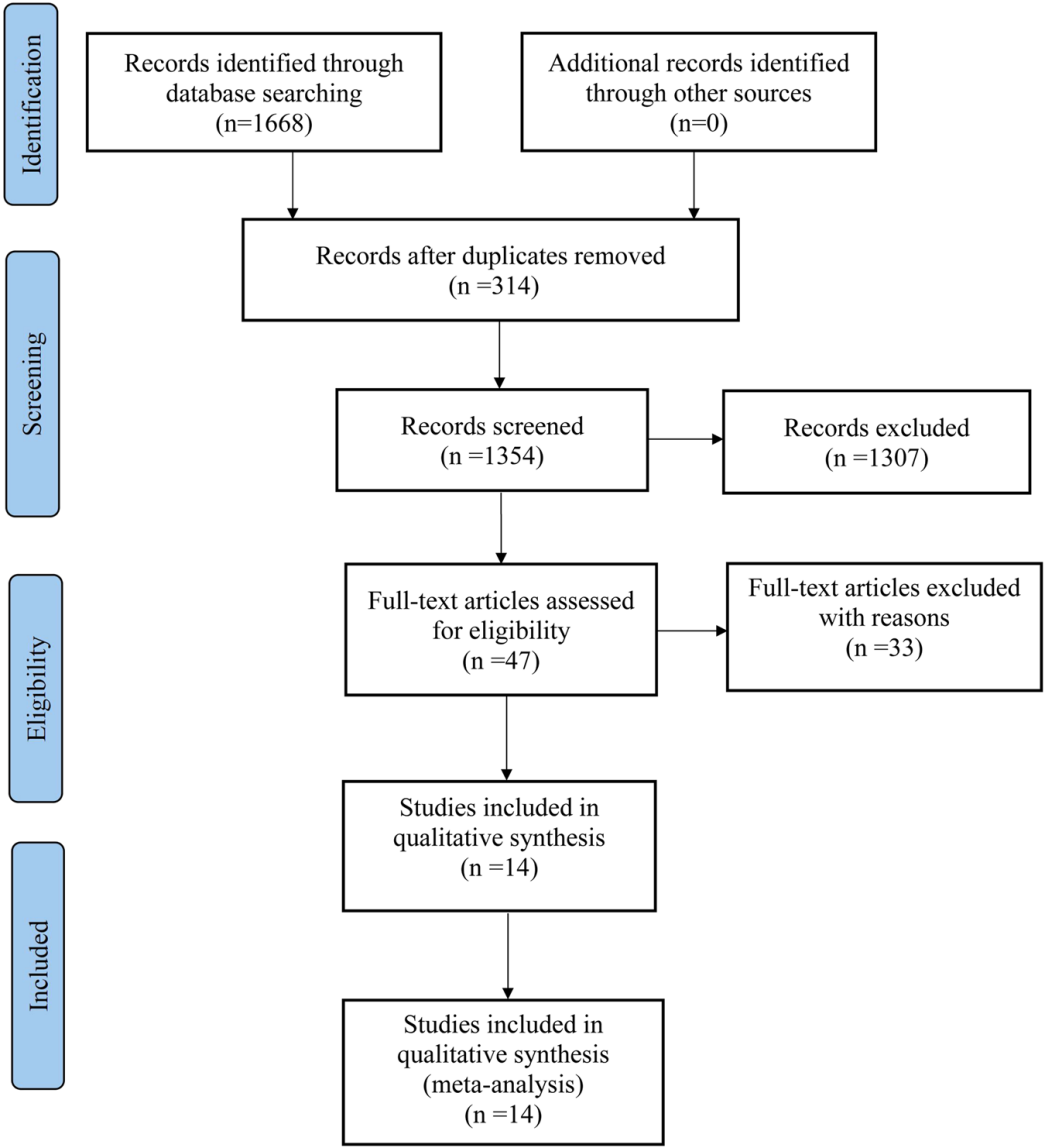
Flow diagram of search and selection of studies

A total of 1668 articles were retrieved; 314 duplicates were removed using Endnote X9 software, and the remaining 1354 articles were used for further analysis. After two researchers read the titles and abstracts of the articles, 1307 articles were excluded, leaving 47 documents. After reading the specific contents of the literature according to the exclusion and inclusion criteria, 14 studies were included in the meta-analysis. A flow diagram of the search and selection of studies is shown in Fig. 1.

### Study characteristics

Fourteen articles [14–27] were finally included in the meta-analysis, which included 4044 patients with type 2 diabetes. In this study, the sample size ranged from 80 to 632 patients. The studies were published between 2004 and 2023. Among the included documents, seven were written in English, and seven were in Chinese. The basic characteristics of the included studies are shown in Table 1.

**Table 1:** Basic characteristics of the included literature

First Author	Year of publication	Country	Study design	No. of recurrent cases	Male		Female		Age(year)		Rate of ASB(%)	Risk factors investigated	NOS score
					ASB	C	ASB	C	ASB	C			
Hasan M Al-Musa	2016	Saudi Arabia	A	246	0	0	31	215	56.3 ± 9.3	49.5 ± 16.7	12.6	①③④	8
Mario Bonadio	2004	Italy	B	176	/	/	/	/	60.1 ± 11.0	63.9 ± 9.7	18.8	①③④⑥	6
A. Ishay	2005	Israel	A	411	0	0	25	386	/	/	6.1	①③④⑤⑩	7
Bashir A. Laway	2021	India	B	400	11	92	59	238	52.81 ± 7.50	52.59 ± 8.25	17.5	①②③④⑤⑦⑩	8
Georgia Matthiopoulou	2023	Greece	B	437	16	154	72	195	/	/	20.1	①②③④⑤⑩	7
Hale Turan	2008	Turkey	B	123	5	27	17	74	60.8 ± 9.5	55.9 ± 10.2	17.8	①②③④⑤⑥⑩	8
Syed Muhammad Jawad Zaid	2020	Pakistan	B	249	/	/	/	/	59.3 ± 3.1	57.7 ± 2.9	19	①③⑩	6
Yunxian Zheng	2004	China	B	80	/	/	/	/	60 ± 10.1	56 ± 11.3	35	①③④⑤⑦⑧⑨	6
Mingwei Chen	2003	China	B	428	/	/	/	/	61 ± 10.2	57 ± 11.5	30	①③④⑤⑦⑧⑨	8
Congqing Miao	2013	China	B	180	21	42	59	58	57.50 ± 9.41	51.41 ± 6.70	44.4	①②③④	7
Mianrong Yu	2005	China	B	300	/	/	/	/	60 ± 10.3	58 ± 9. 6	25	①③④⑤⑦⑧⑨	7
Dongjuan He	2007	China	B	268	/	/	/	/	61.2 ± 10.2	56.1 ± 11.3	25.4	①③④⑤⑦⑧⑨	7
Xiaocen Kong	2016	China	B	632	50	257	99	226	71.46 ± 6.71	69.84 ± 6.62	23.58	①②③④	6
Yiwen Zhao	2012	China	B	114	7	22	23	62	60.3 ± 9.6	56.4 ± 10.3	26.32	①③④	8

A: cohort study, B: case–control study

①age, ②gender, ③ Duration of diabetes, ④HbA1c, ⑤BMI, ⑥Glomerular filtration rate, ⑦hypertension, ⑧hyperlipidemia, ⑨neuropathy, ⑩albuminuria

### Risk of bias assessment

Literature quality evaluation was scored using the NOS scale, with 9 being the highest score and greater than or equal to 6 being a good-quality cohort study or case-control study. In the scoring results, All case-control studies meet the requirements of “Adequate definition of cases,” “Representativeness of the cases,” The conditions of “ascent of exposure,” and “same method of ascent for cases and controls” are not met by some cohort studies. Please refer to Table 2 for a detailed quality evaluation of the cohort studies. Please refer to Table 3 for a detailed quality evaluation of the case-control studies.

**Table 2:** Result of quality assessment using the Newcastle-Ottawa Scale for the cohort study

Study	Selection				Comparability	Outcome			NOS score
	Representativeness of the exposed cohort	Selection of the non exposed cohort	Ascertainment of exposure	Demonstration that outcome of interest was not present at start of study	Comparability of cohorts on the basis of the design or analysis	Assessment of outcome	Follow-up long enough for outcomes to occur	Adequacy of follow up of cohorts	
Hasan M Al-Musa,2016	☆	☆	☆	☆	☆	☆	☆	☆	8
A. Ishay,2005	☆		☆	☆	☆	☆	☆	☆	7

**Table 3:** Result of quality assessment using the Newcastle-Ottawa Scale for the case–control studies

Study	Selection				Comparability	Exposure			NOS score
	Adequate definition of cases	Representativeness of the cases	Selection of Controls	Definition of Controls	Comparability of cases and controls on the basis of the design or analysis	Ascertainment of exposure	Same method of ascertainment for cases and controls	Non-Response rate	
Mario Bonadio (2004)	☆	☆		☆	☆	☆	☆		6
Bashir A. Laway (2021)	☆	☆	☆	☆	☆☆	☆	☆		8
Georgia Matthiopoulou (2023)	☆	☆	☆	☆	☆	☆	☆		7
Hale Turan (2008)	☆	☆	☆	☆	☆☆	☆	☆		8
Syed Muhammad Jawad Zaid (2020)	☆	☆		☆	☆	☆	☆		6
Yunxian Zheng (2004)	☆	☆		☆	☆	☆	☆		6
Mingwei Chen (2003)	☆	☆	☆	☆	☆☆	☆	☆		8
Congqing Miao (2013)	☆	☆	☆	☆	☆	☆	☆		7
Mianrong Yu (2005)	☆	☆	☆	☆	☆	☆	☆		7
Dongjuan He (2007)	☆	☆	☆	☆	☆	☆	☆		7
Xiaocen Kong (2016)	☆	☆		☆	☆	☆	☆		6
Yiwen Zhao (2012)	☆	☆	☆	☆	☆☆	☆	☆		8

## Meta-analysis results

### Prevalence of asymptomatic bacteriuria in patients with type 2 diabetes mellitus

**Fig. 2:**
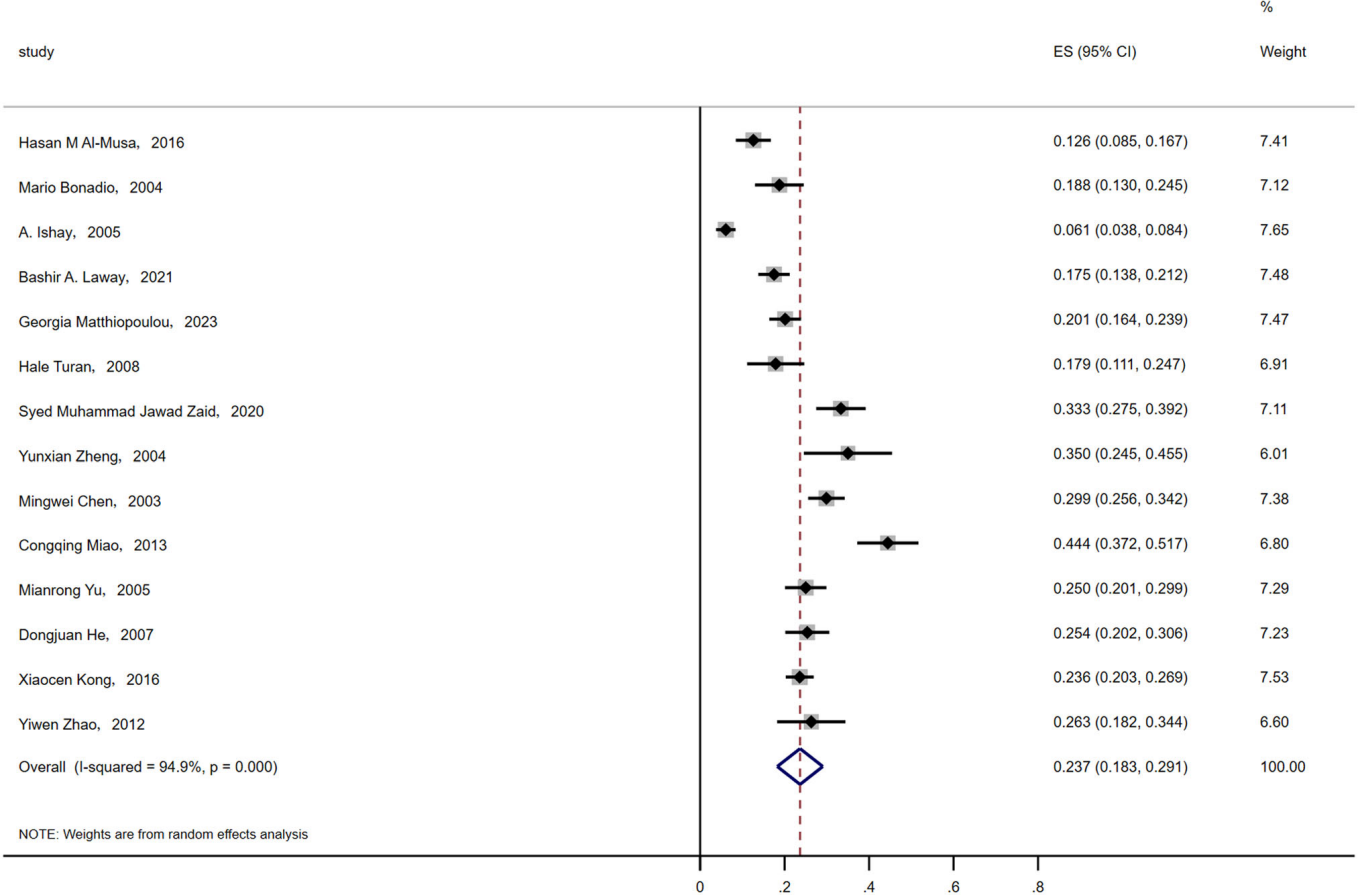
Incidence of ASB in T2DM

A meta-analysis identified 910 patients with T2DM complicated with ASB and 3494 patients without asymptomatic bacteriuria. The incidence of ASB in T2DM was 23.7% (*P* < 0.001) Fig. 2.

### Risk factors of asymptomatic bacteriuria in type 2 diabetes mellitus

A meta-analysis showed that age, female sex, duration of diabetes, HbA1c, hypertension, hyperlipidemia, neuropathy, and proteinuria were risk factors for asymptomatic bacteriuria in patients with T2DM.

#### Age

**Fig. 3:**
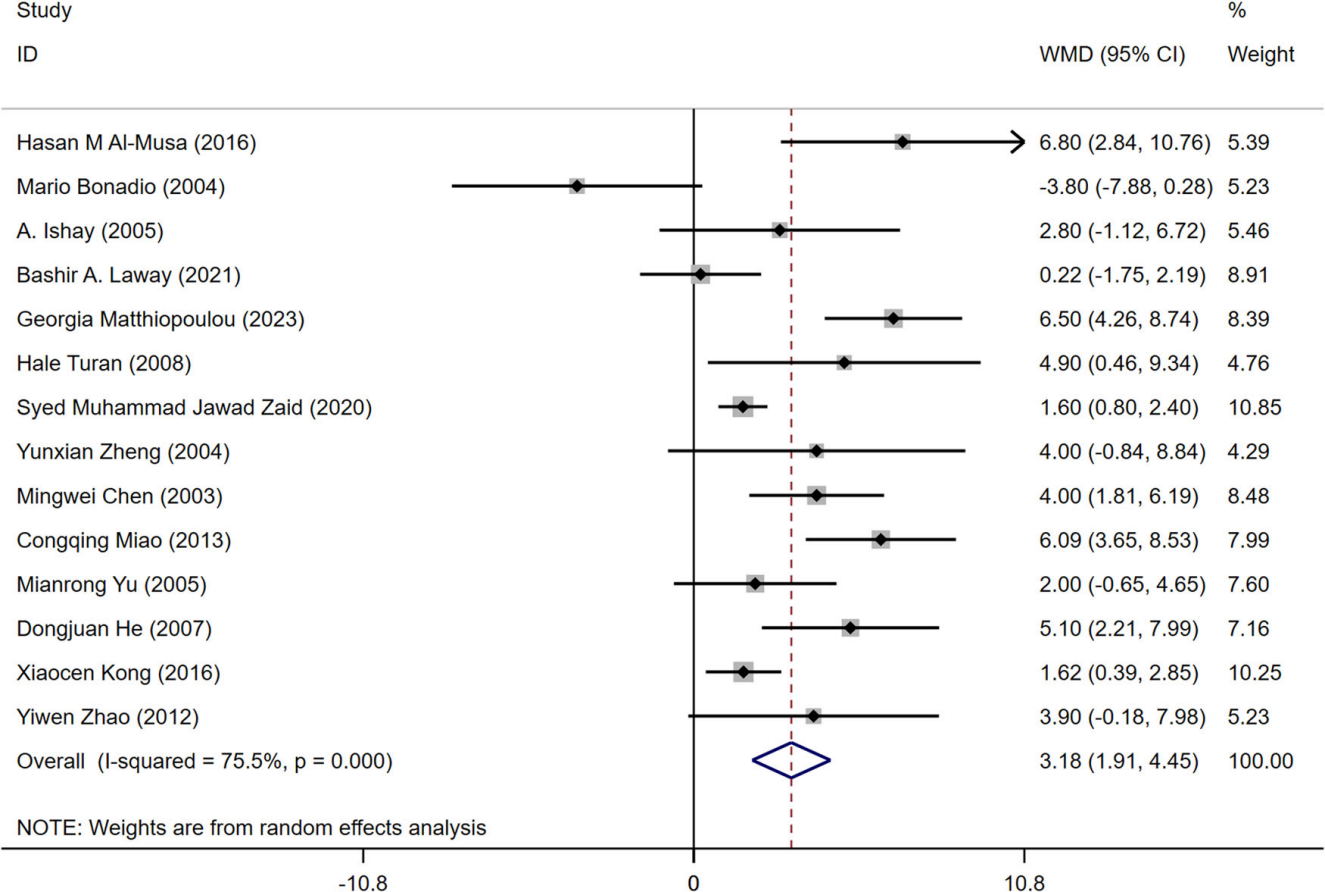
Meta-analysis of age as a risk factor of ASB

**Fig. 4:**
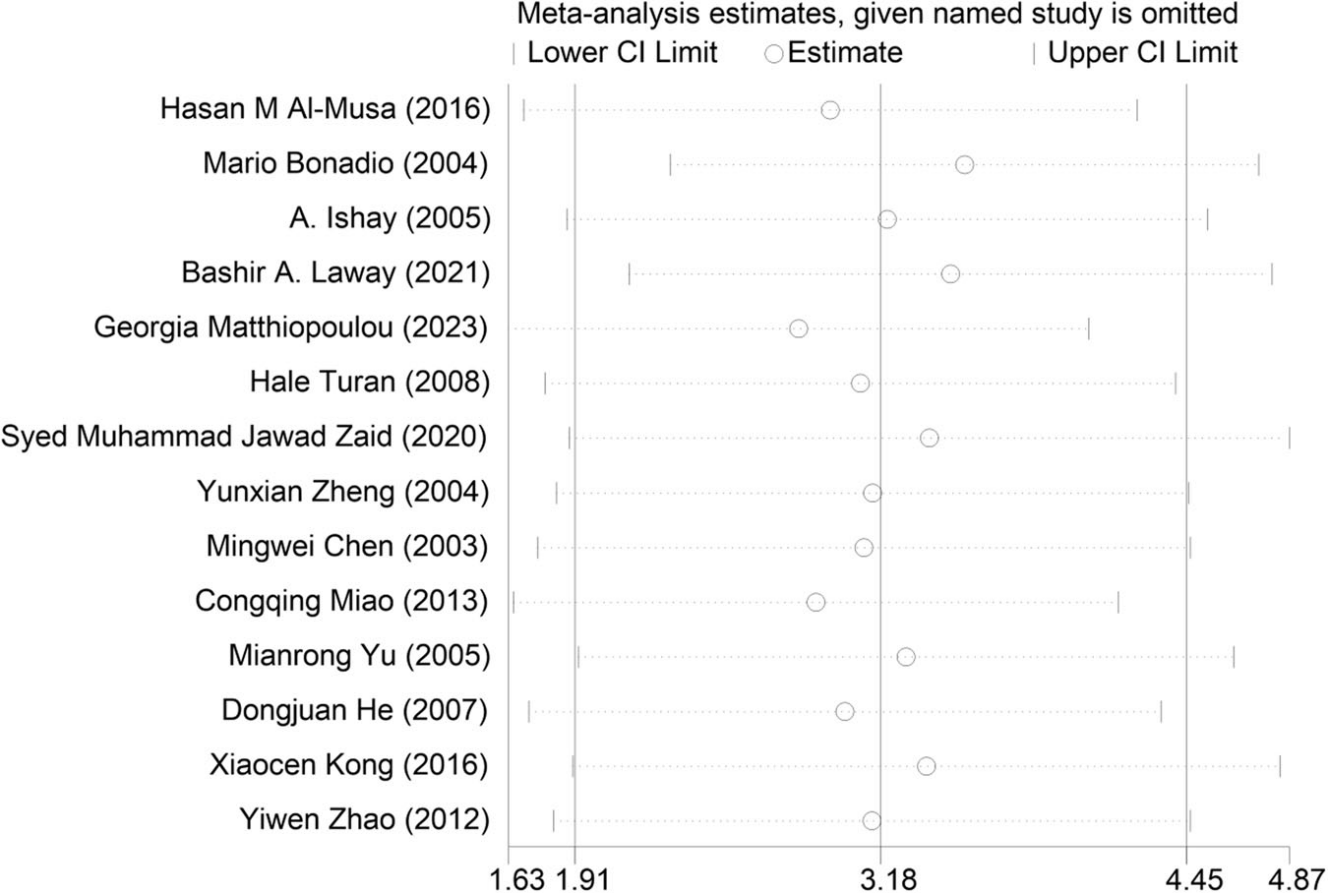
Sensitivity analysis of the meta-analysis of age as a risk factor for ASB

**Fig. 5:**
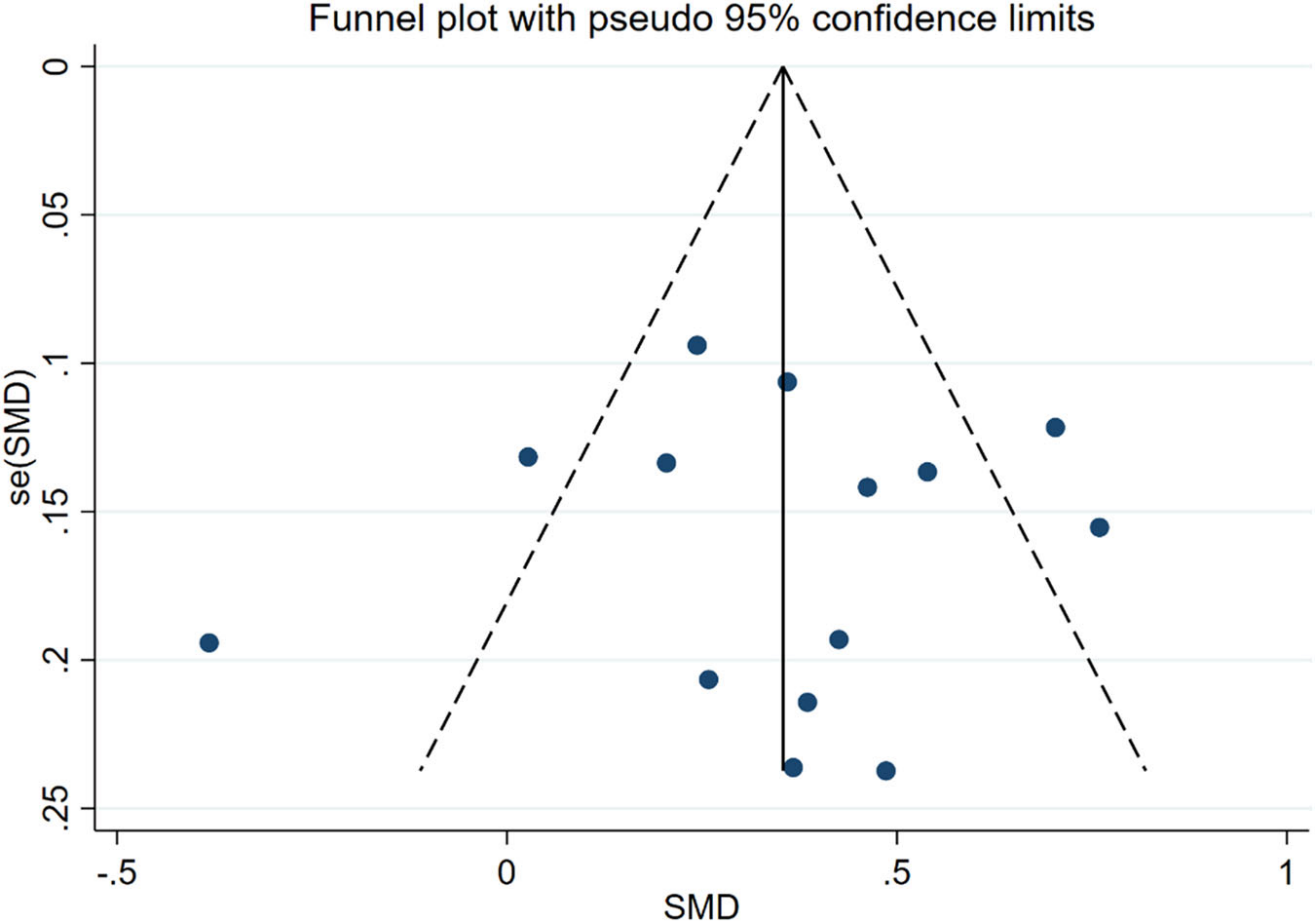
Funnel plots of the meta-analysis of age as a risk factor for ASB

The 14 articles [14–27] included age-related data. Among them, 13 documents [15–27] showed that the patients with ASB were older than those in the control group, and one article [14] showed that the control group was older than the ASB group. Because of heterogeneity, the random effects model showed that older patients with T2DM were more likely to get asymptomatic bacteriuria (WMD = 3.18, 95% CI [1.91, 4.45], *I*^2^ = 75.5%, *P* < 0.001) Fig. 3. Because of the high heterogeneity of the studies, we conducted a sensitivity test and subgroup analysis (divided into foreigners and Chinese people). The sensitivity test showed that the results were stable, and the heterogeneity of the subgroup analysis did not change significantly (Fig. 4). These results may be attributed to significant ethnic differences between the populations of all the articles, resulting in high heterogeneity. The funnel diagram was roughly symmetrical, showing no publication bias in the included literature (Fig. 5).

#### Female sex

**Fig. 6:**
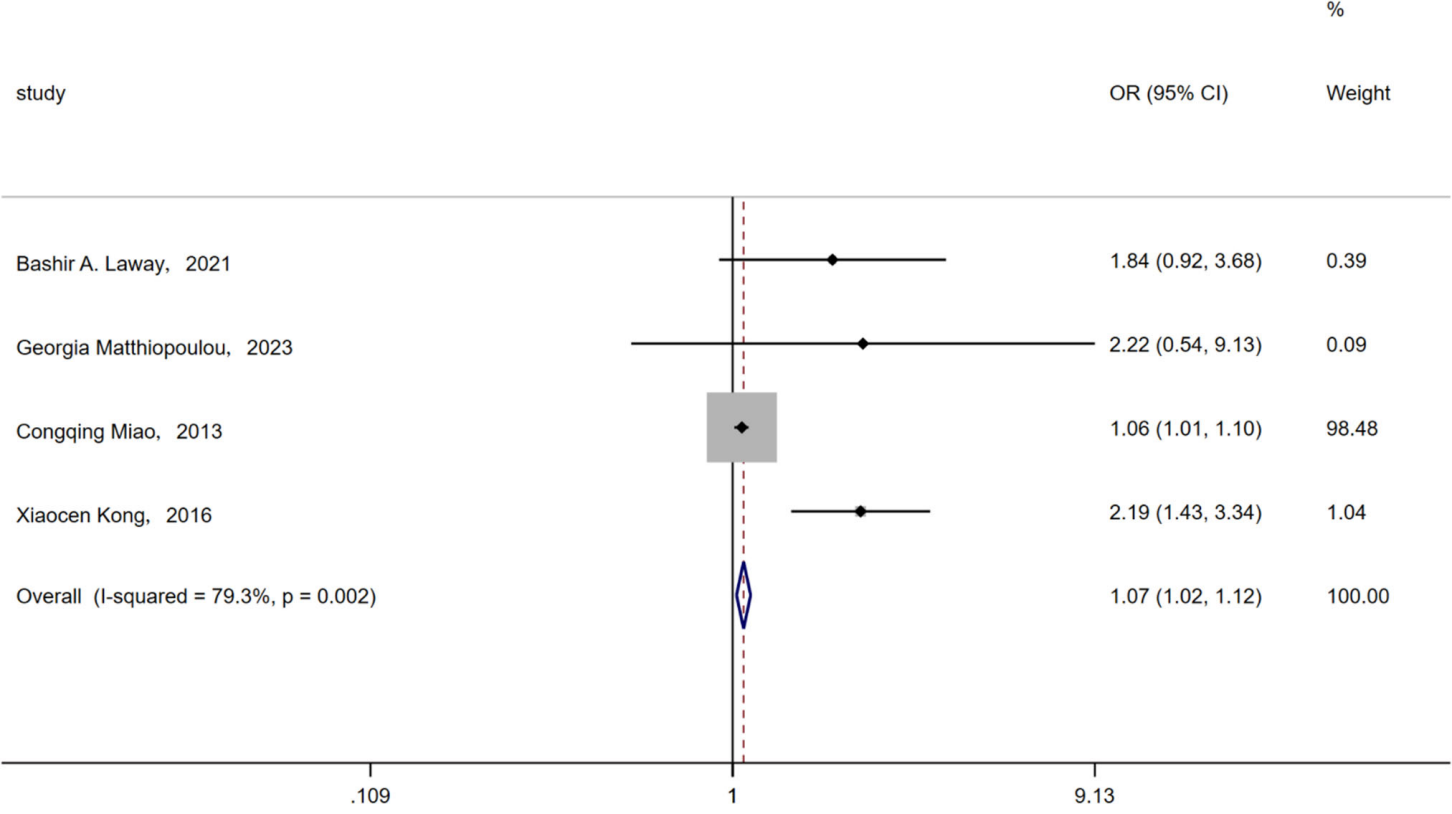
Meta-analysis of female gender as a risk factor of ASB

There were four articles [16, 18, 24, 25] that included female sex as a variable. The probability of ASB in female patients with T2DM in four studies was higher than that in male patients. A random effects model was used to analyze heterogeneity. The results showed that the female sex was a risk factor for ASB in patients with T2DM (OR = 1.07, 95% CI [1.02, 1.12], *I*^2^ = 79.3%, *P* = 0.002) (Fig. 6). Owing to the small number of articles, subgroup analysis was not performed. Among them, the article by Congqing Miao [25] may be the source of heterogeneity.

#### Duration of type 2 diabetes mellitus

**Fig. 7:**
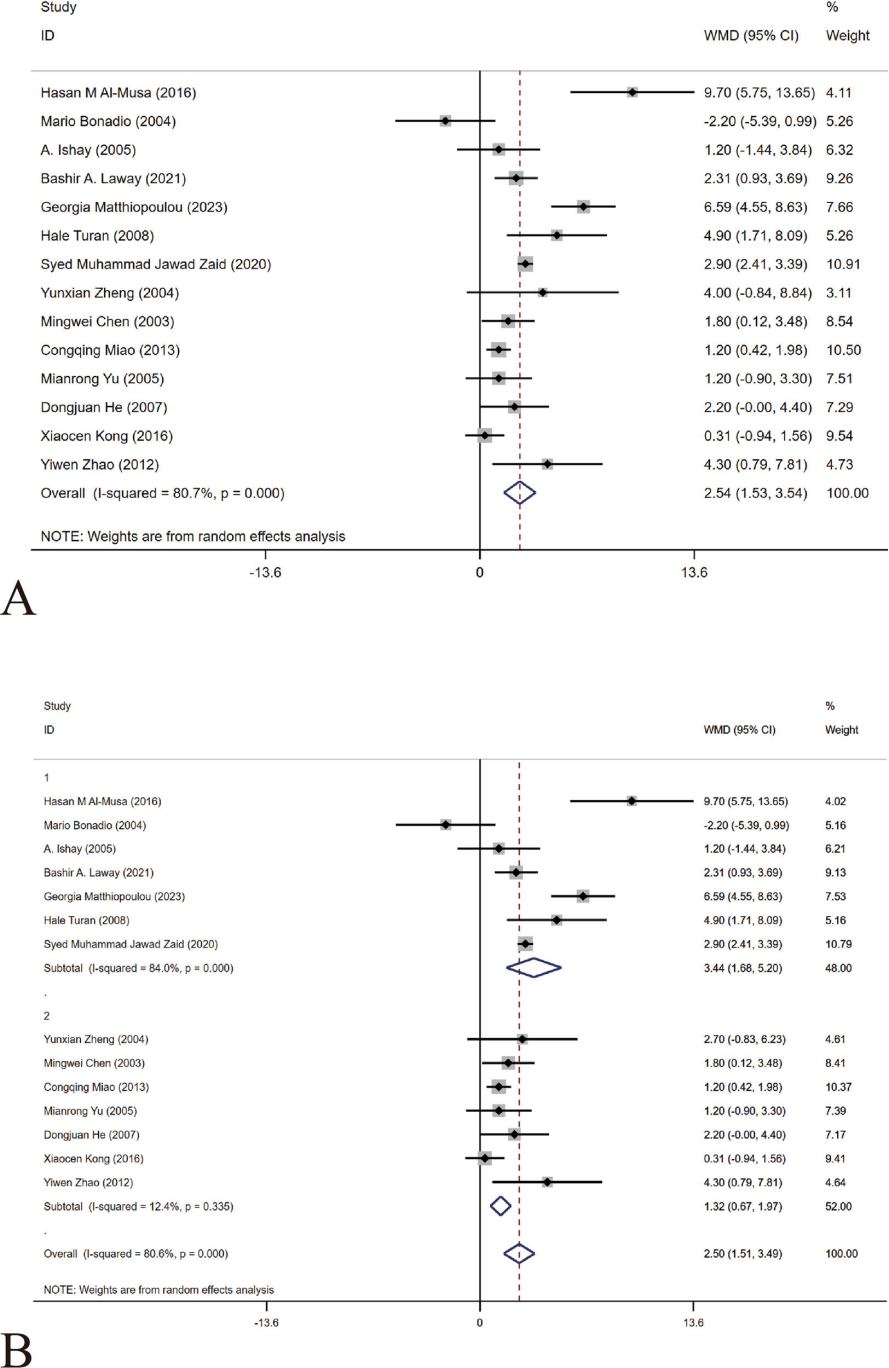
Meta-analysis (included subgroup analysis) of the duration of T2DM as a risk factor of ASB. **A** The results of meta-analysis (all articles included) of the duration of T2DM as a risk factor of ASB. **B** The results of subgroup analysis (subgroups are classified for China or foreign countries according to the countries where the articles have been published) of duration of T2DM as a risk factor of ASB

**Fig. 8:**
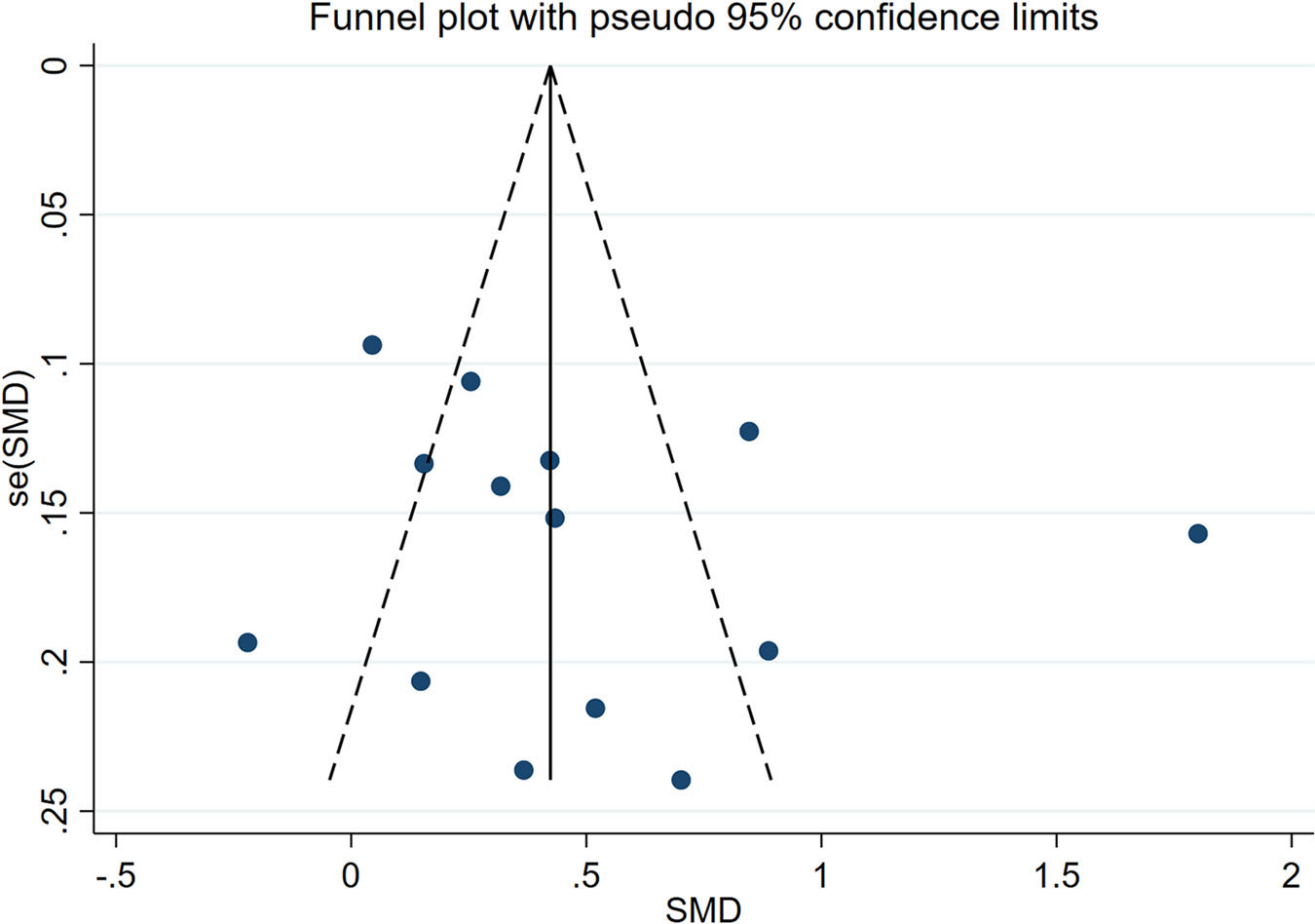
Funnel plots of the meta-analysis of duration of T2DM as a risk factor for ASB

The included 14 articles [14–27] included diabetes disease duration as a variable. Only one article [14] showed that a prolonged illness time negatively correlated with ASB in patients with T2DM. Because of heterogeneity, a random effects model was used. The results showed that a prolonged illness duration was a risk factor for ASB (WMD = 2.54, 95% CI [1.53, 5.43], *I*^2^ = 80.7%, *P* < 0.001) Fig. 7A. We conducted a sensitivity analysis and found that the results were robust for this study. The results of subgroup analysis (China and foreign countries) showed that the heterogeneity of Chinese studies was small (WMD = 3.44, 95% CI [1.68, 5.20] *I*^2^ = 84%, *P* < 0.001), and the primary source of heterogeneity was foreign articles (WMD = 2.50, 95%CI [1.51, 3.49] *I*^2^ = 12.4%, *P* < 0.001) Fig. 7B. The funnel diagram was roughly symmetrical, indicating no publication bias (Fig. 8).

#### HbA1c

**Fig. 9:**
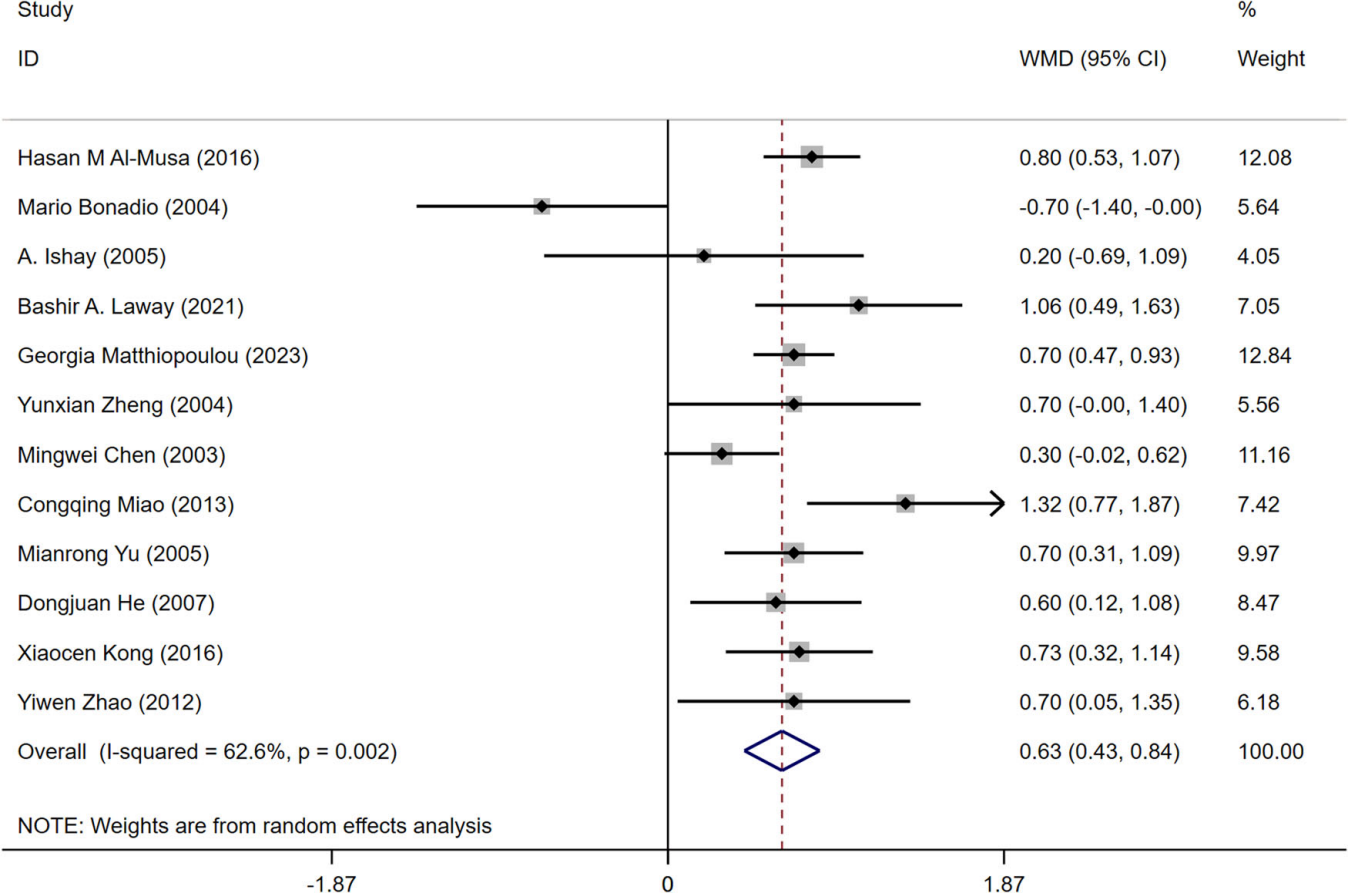
Meta-analysis of HbA1c as a risk factor of ASB

**Fig. 10:**
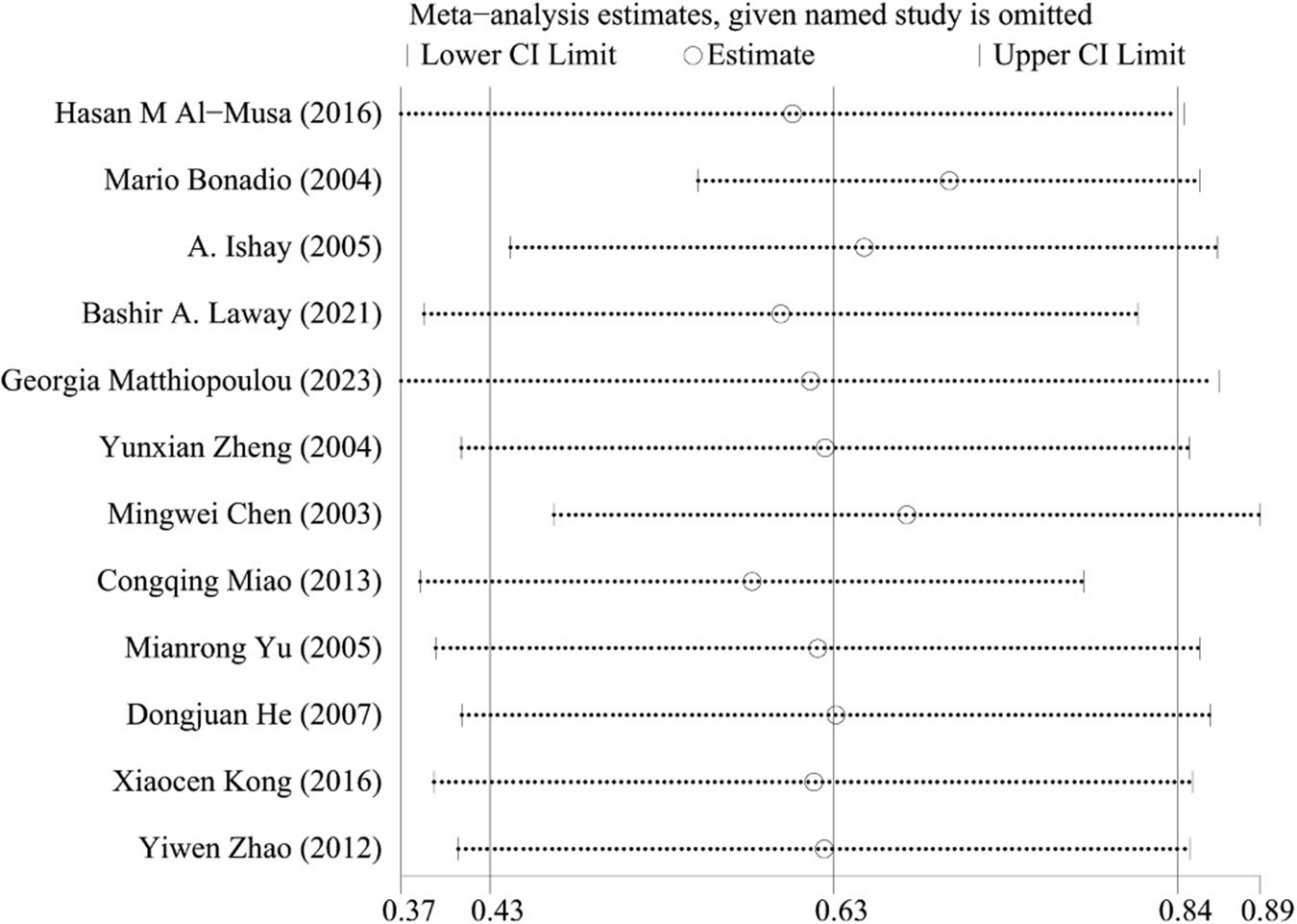
Sensitivity analysis of the meta-analysis of Hb1Ac as a risk factor for ASB

**Fig. 11:**
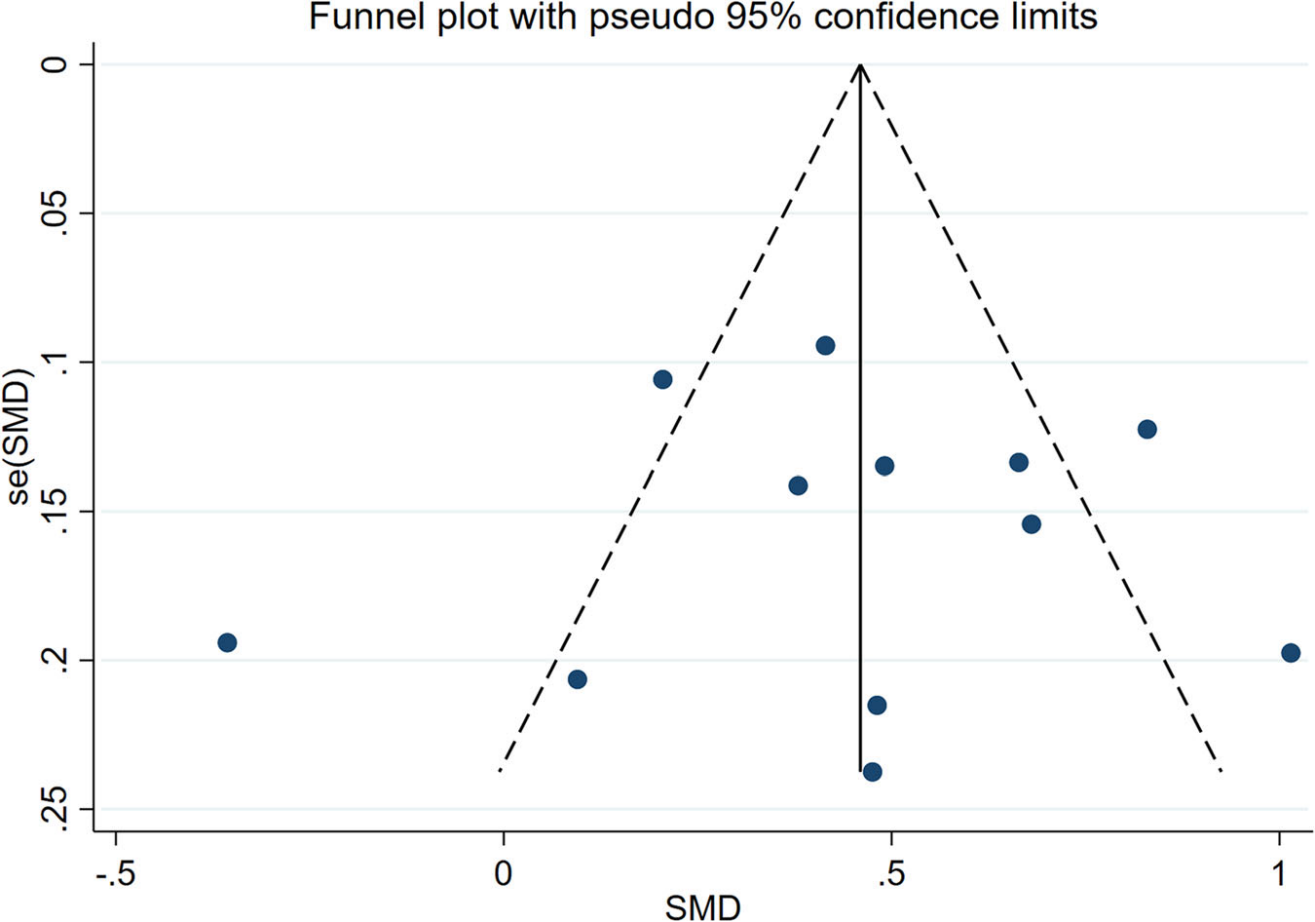
Funnel plots of the meta-analysis of HbA1c as a risk factor for ASB

A total of 12 articles [14–18, 21–25, 27] mentioned HbA1c levels. One study showed that HbA1c levels were negatively correlated with ASB levels in patients with T2DM. A total of 805 patients with T2DM complicated by ASB and 2,867 patients without ASB were included. Because of the heterogeneity, the random-effect model was selected. The results showed that HbA1c was a risk factor for ASB in T2DM (WMD = 0.63, 95% CI [0.43, 0.84], *I*^2^ = 62.6%, *P* < 0.001) (Fig. 9). We conducted a sensitivity analysis, and the results were relatively stable (Fig. 10). The funnel diagram was roughly symmetrical, indicating no publication bias Fig. 11.

#### Body Mass Index (BMI)

**Fig. 12:**
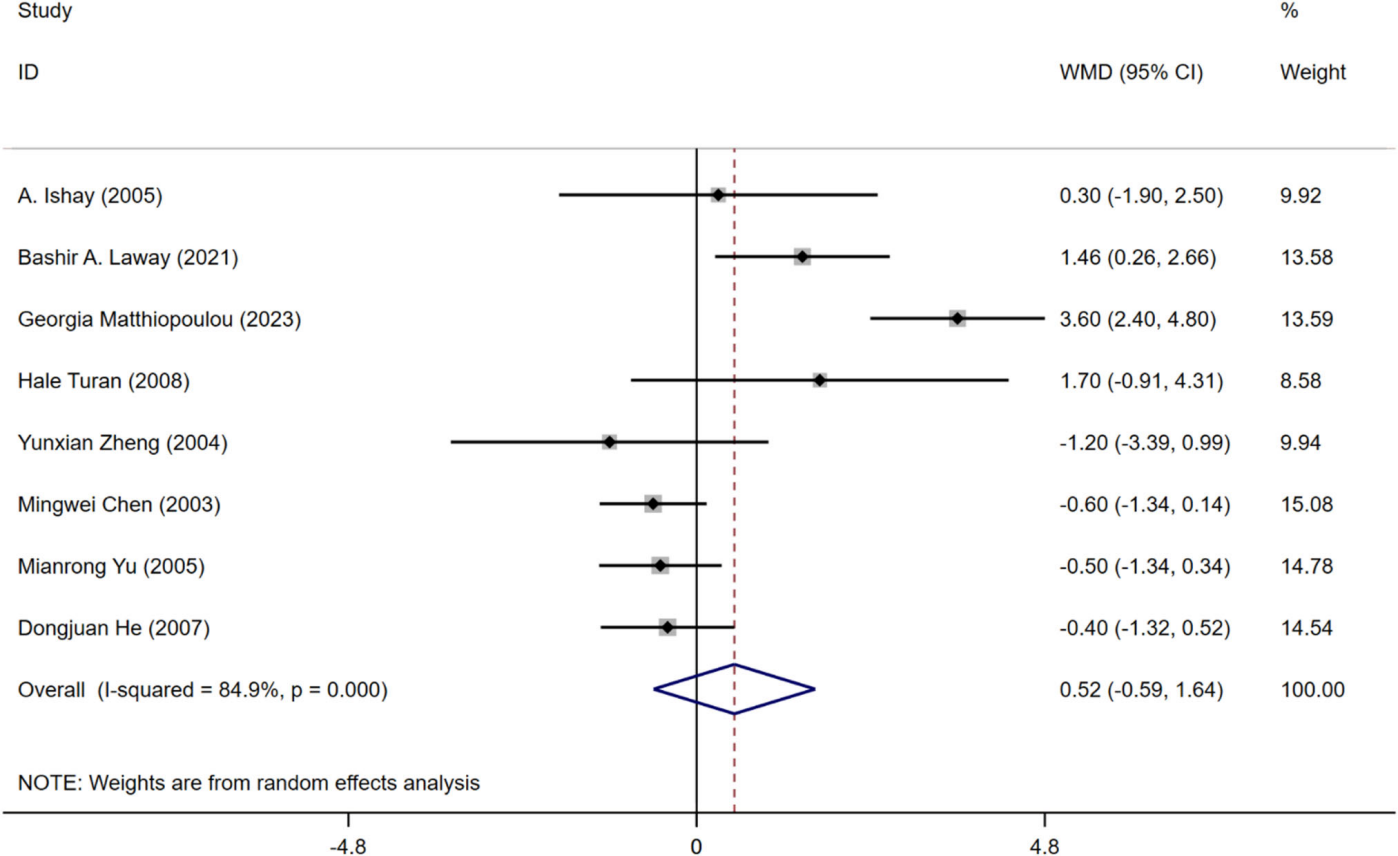
Meta-analysis of BMI as a risk factor of ASB

Eight articles [16–19, 21–23, 26] mentioned BMI. Among them, four articles [16–19] reported a positive correlation of BMI with ASB, and four articles [21–23, 26] reported that BMI was negatively correlated with ASB. A total of 504 patients with ASB and 1943 patients without ASB were included. Because of the heterogeneity, the random-effect model was selected. A meta-analysis of random effects showed that BMI was not a risk factor for ASB in patients with T2DM (WMD = 0.52, 95%CI [-0.59, 1.64], *I*^2^ = 84.9%, *P* = 0.359) (Fig. 12).

#### Glomerular filtration rate

**Fig. 13:**
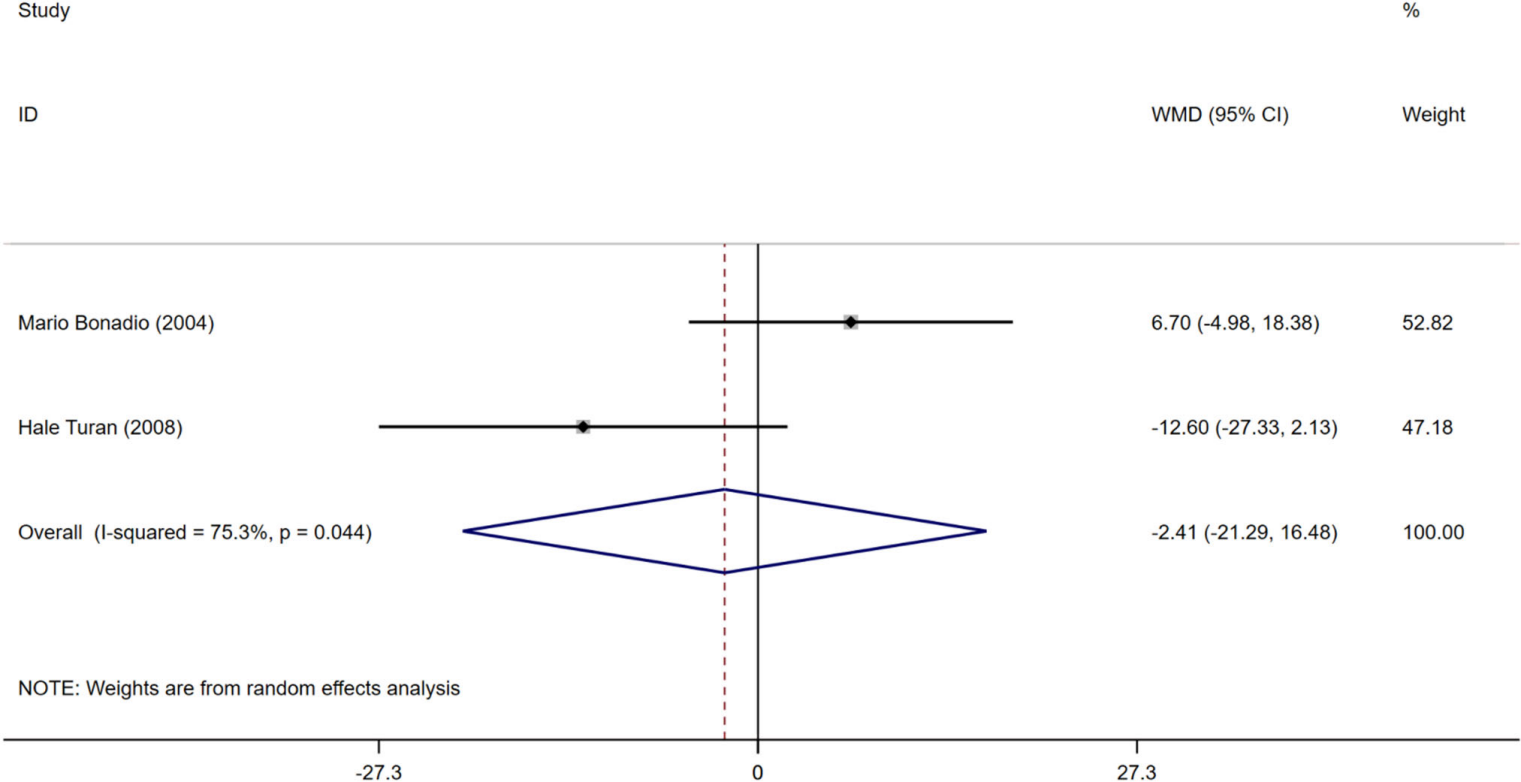
Meta-analysis of eGFR as a risk factor of ASB

Two articles [14, 19] reported data on the glomerular filtration rate. The two articles reported different conclusions regarding whether the glomerular filtration rate is a risk factor for ASB. Meta-analysis of random effects showed that the glomerular filtration rate was not a risk factor for ASB in patients with T2DM (WMD = −2.41, 95%CI [−21.29, 16.48], *I*^2^ = 75.3%, *P* = 0.803) (Fig. 13).

#### Type 2 diabetes mellitus complicated with other diseases

##### Type 2 diabetes mellitus complicated with hypertension

**Fig. 14:**
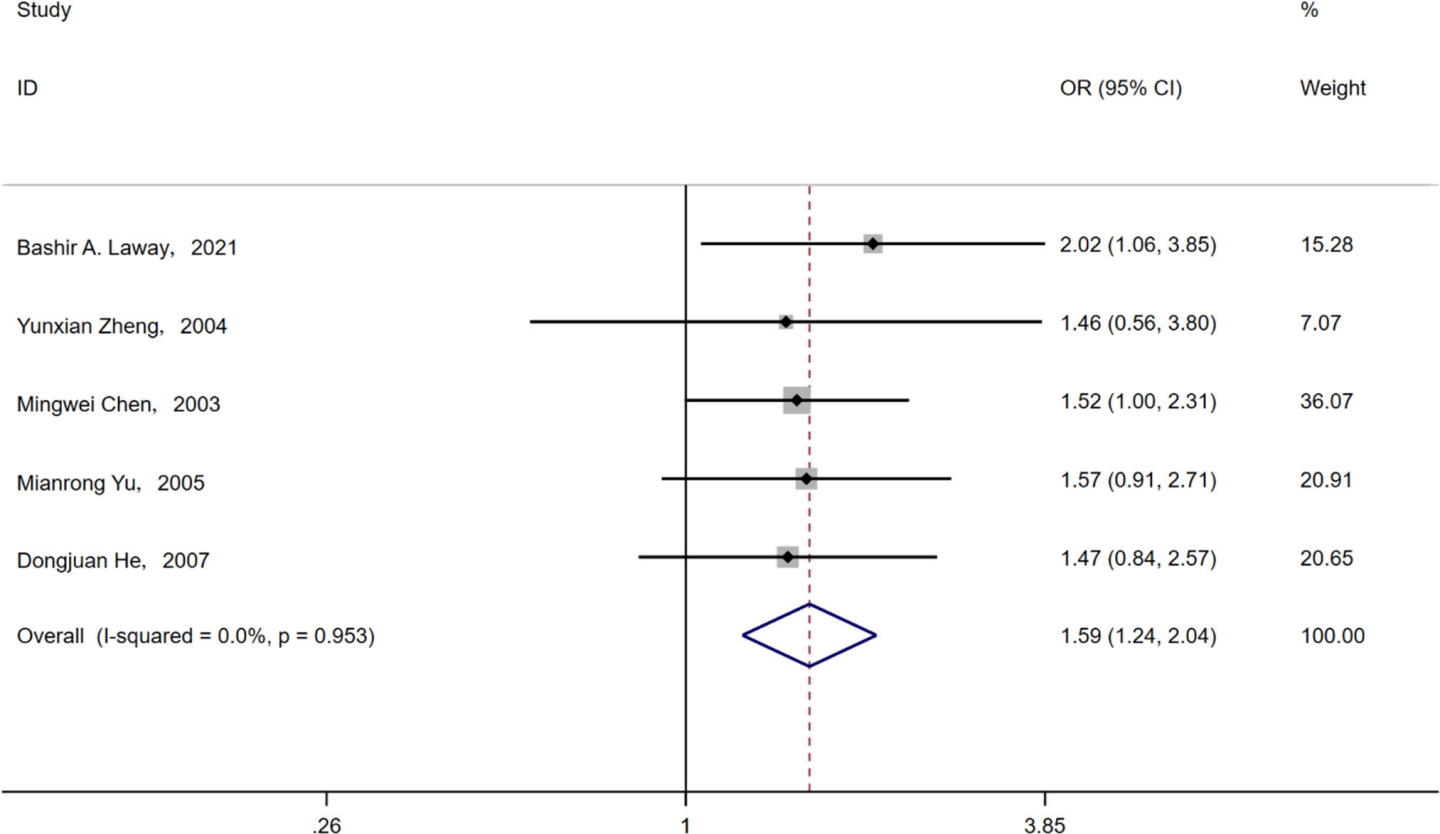
Meta-analysis of hypertension as a risk factor of ASB

Five articles [17, 21–23, 26] mentioned cases of diabetes complicated with hypertension, and five articles [17, 21–23, 26] reported that hypertension was positively associated with the incidence of ASB. Because of the small heterogeneity, the fixed model was selected for meta-analysis. It was concluded that hypertension alongside T2DM was a risk factor for ASB (OR = 1.59, 95% CI [1.24, 2.04], *I*^2^ = 0%, *P* < 0.001) (Fig. 14).

##### Type 2 diabetes mellitus complicated with hyperlipidemia

**Fig. 15:**
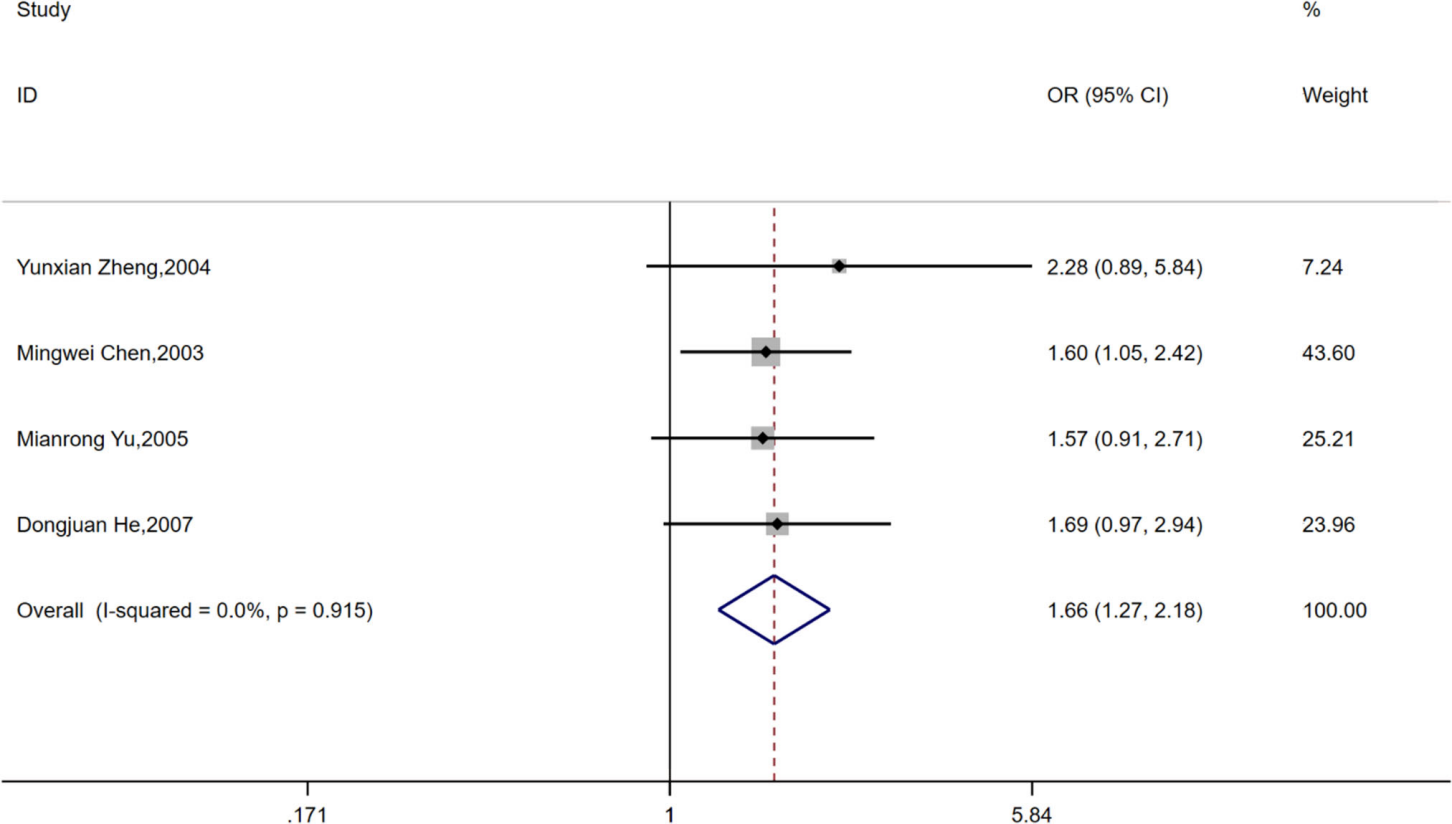
Meta-analysis of Hyperlipidemia as a risk factor of ASB

Four articles [21–23, 26] mentioned the situation of diabetes complicated by hyperlipidemia, and all four articles [21–23, 26] thought that hyperlipidemia was positively related to the incidence of ASB. Because of the small heterogeneity, a fixed model was selected for meta-analysis, and it was concluded that patients with hyperlipidemia at the same time were the risk factors for obtaining ASB (OR = 1.66, 95% CI [1.27, 2.18], *I*^2^ = 0%, *P* < 0.001) (Fig. 15).

##### Type 2 diabetes mellitus complicated with neuropathy

**Fig. 16:**
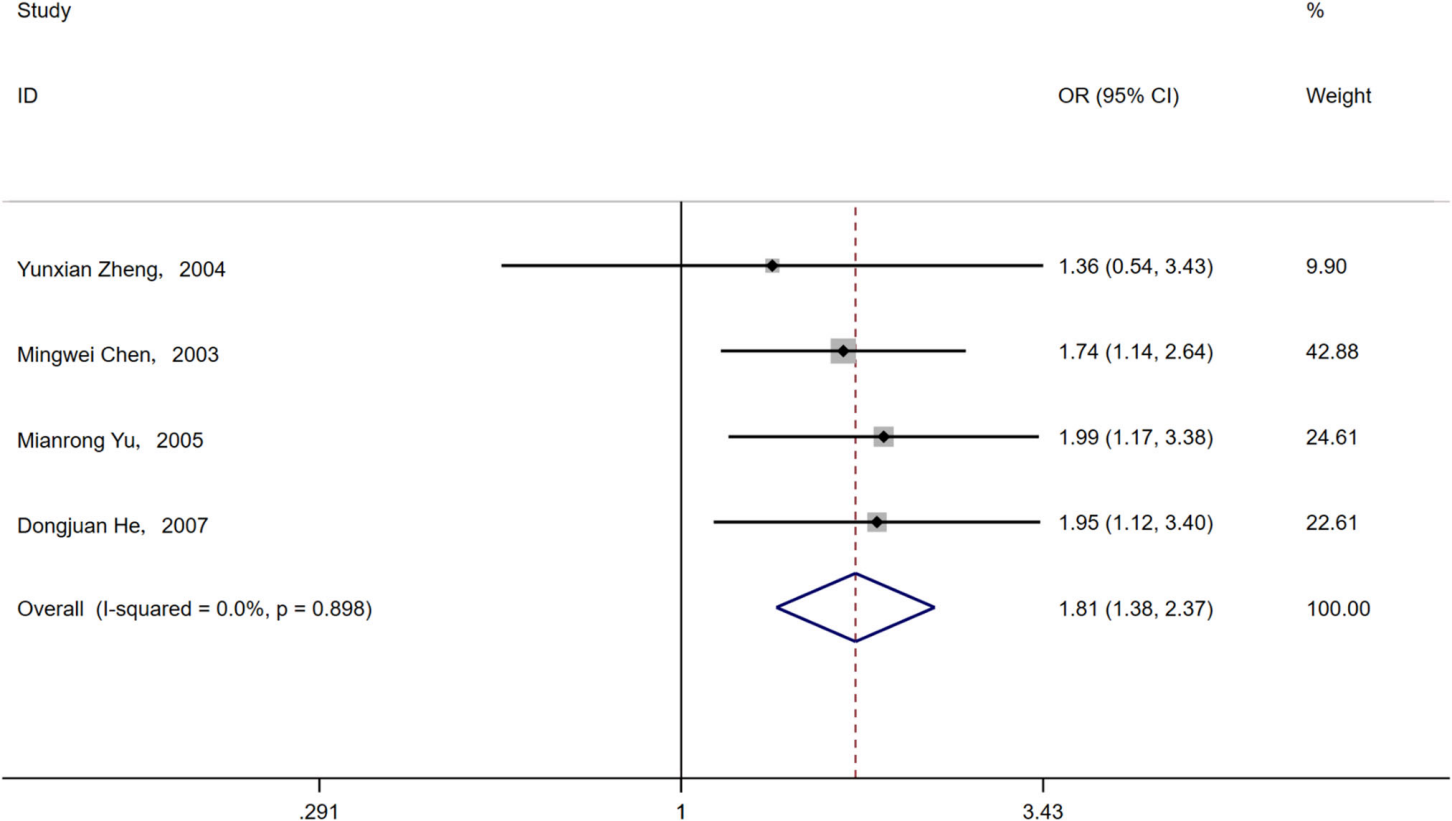
Meta-analysis of Neuropathy as a risk factor of ASB

Four articles [21–23, 26] mentioned diabetes complicated with neuropathy, and patients with neuropathy in four articles [21–23, 26] were reportedly more likely to develop ASB. Because of the small heterogeneity, a fixed model was selected for meta-analysis, and it was concluded that diabetes complicated with neuropathy was a risk factor for ASB (OR = 1.81, 95% CI [1.38, 2.37], *I*^2^ = 0%, *P* < 0.001) (Fig. 16).

#### Albuminuria

**Fig. 17:**
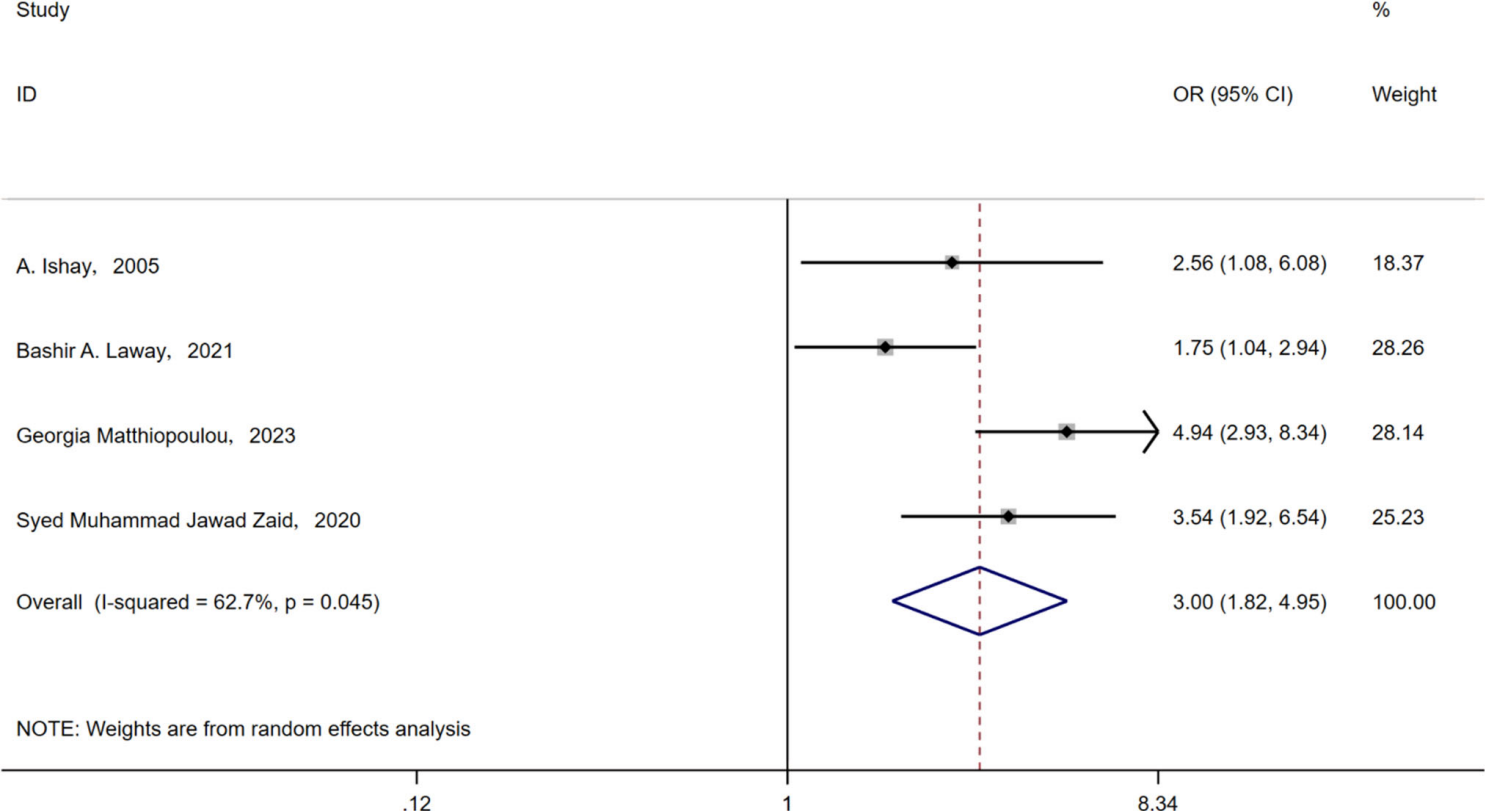
Meta-analysis of proteinuria as a risk factor of ASB

Four articles [16–18, 20] mentioned proteinuria, and all four articles [16–18, 20] suggested that patients with proteinuria were more likely to suffer from ASB. A meta-analysis with a random model showed that proteinuria was one of the risk factors for ASB (OR = 3.00, 95% CI [1.82, 4.95], *I*^2^ = 62.7%, *P* < 0.001) (Fig. 17).

## Discussion

### Incidence of asymptomatic bacteriuria

Fourteen studies included in this meta-analysis showed that the incidence of ASB in patients with T2DM was 23.7%, higher than that reported by Guide [11] and Zhanel [28]. This may be because previous research data were obtained before 1991. With the development of medical care and society, the detection rate may increase because of the development of science and technology [29]. In most of the studies from developing countries, due to the low level of health development, patients’ own management efforts, and insufficient awareness of disinfection and cleanliness, the incidence increased. Changes in the global demographic structure may also be one of the reasons for the increase in the prevalence rate [30]. The specific reasons for the rising prevalence rates require further investigation.

### Correlation between asymptomatic bacteriuria and patients’ personal characteristics (age, female gender, BMI)

Age was a risk factor for ASB. The decline in self-function in such patients, coupled with diabetes, leads to an imbalance in the internal environment of patients [31]. The metabolism of sugar, fat, and protein in the human body affects the immune system of the human body [32]. The human body’s ability to fight pathogens decreases, the patient’s urethral mucosal defense barrier function decreases, and pathogens are more likely to invade, increasing the risk of ASB infection [13, 33]. The urethra of women is short, close to the anus, and the colonization rate of pathogens around the urethra is high. The glycogen content of vaginal epithelium decreases due to diabetes, the pH value increases, the ecological environment of vaginal lactic acid bacteria is destroyed, the flora is unbalanced, and it is easily infected. Some female patients were menopausal. Degenerative changes in the urethral mucosa in such patients lead to an increase in the incidence of ASB [34, 35]. Estrogen is a protective factor against urinary tract infection in females, which can increase the gene expression of proteins and antimicrobial peptides and reduce the bacterial titer in cells. However, the incidence of ASB increases with the decrease of estrogen in menopausal women [36, 37]. In this study, it was not proven that BMI is a risk factor for asymptomatic bacteriuria in patients with type 2 diabetes mellitus, and differences in race and measurement tools may have affected the analysis. However, BMI may be a risk factor for ASB because patients with higher BMI have more body fat and higher plasma osmotic pressure, which leads to lower phagocytosis of pathogenic bacteria by white blood cells [38]. Simultaneously, the immune-regulated pro-inflammatory cytokines involved in obesity can also lead to systemic inflammation, thus reducing patients’ immunity and increasing the probability of bacterial infection. An increase in BMI causes insulin resistance and increases the incidence of infection in T2DM [39].

For patients with these characteristics, clinicians should observe the symptoms related to urine and the urinary system. For female patients with T2DM, awareness of self-cleaning should be widely popularized, and behaviors such as urination immediately after sexual activity, changing and cleaning underwear on time, improving immunity, and using immune enhancers appropriately, should be encouraged.

### Correlation between asymptomatic bacteriuria and patients’ diseases (course of disease, HbA1c, glomerular filtration rate, proteinuria)

The course of the disease and HbA1c are risk factors for ASB in patients with T2DM. Studies have shown that the possibility of ASB infection increases by 1.9 times every 10 years in patients with diabetes [40]. Long-term abnormal blood sugar levels lead to increased osmotic pressure in body fluids, disordered metabolism of immune cells, and decreased chemotaxis, phagocytosis, and bactericidal ability. Owing to the accumulation of advanced glycation end products (AGEs) in the urinary tract mucosa, there is an endogenous “invisible injury” in the mucosa without destruction, and the mucosal barrier and protection are weakened, causing ASB [41]. HbA1c is a biological index for detecting diabetes, which can reflect patients’ blood sugar control and insulin resistance in the past three months [42]. Because patients in developing countries do not fully understand and manage their disease, a high glucose state can damage B lymphocytes, which are stimulated by antigens to produce antibodies, mediate humoral immunity, and change the IgG level in patients with diabetes. The IgG levels of patients were high in the reported studies. IgG is an immunoglobulin secreted by plasma cells in the bladder and urethral walls. Can make a smooth colony change into a rough colony and increase the incidence of ASB [43, 44].

Diabetic nephropathy (DN) is one of the leading causes of end-stage renal disease worldwide [45]. Glomerular filtration rate was used to evaluate renal function [46]. Patients with diabetes and caregivers often pay attention only to changes in blood sugar levels. Irreversible damage often occurs when patients observe their urine changes (foam urine) and edema of both lower limbs. In addition, urinary microalbumin, a small molecular weight protein, cannot pass through the glomerular filtration membrane. Due to hyperglycemia, protein glycosylation of the glomerular basement membrane increases the protein filtration volume, and the glomerulus cannot completely reabsorb it, eventually leading to urinary excretion and proteinuria [47, 48]. Because many immunoglobulins are lost from urine, low plasma albumin affects antibody formation, and abnormal metabolism in patients with diabetes increases the possibility of infection in proteinuria patients [49–51]. Studies have shown that patients with diabetes complicated with proteinuria have an increased possibility of kidney injury [51]. This study failed to verify whether the glomerular filtration rate is a risk factor for ASB in patients with T2DM, which may be associated with the small number of original documents included in this index.

For patients with diabetes, symptom management is critical so that patients’ blood sugar can reach an ideal level, effectively preventing the emergence of acute and chronic complications of diabetes. The management of DN is not limited to drugs and other treatments; lifestyle interventions are also important measures to promote health. Healthy eating habits, weight control, proper exercise, smoking cessation, and good sleep habits can effectively delay the progression of kidney disease in patients with diabetes.

### Correlation between asymptomatic bacteriuria and patients complicated with diseases (hypertension, hyperlipidemia, neuropathy)

The inflammatory corpuscles endothelin-1, angiotensin-2, and aldosterone are activated in patients with diabetes mellitus complicated by hypertension. NLRP3 (NOD [nucleotide oligomerization domain]-like receptor family pyrin domain containing 3) activation and inflammasome assembly is triggered through either ATP-induced K^+^ efflux or generation of reactive oxygen species, which are considered classical activators of the NLRP3 inflammasome during hypertension [52]. Asymptomatic bacteriuria may be caused by the activation of inflammatory corpuscles by active oxidants in patients. Adiponectin content in hyperlipidemic patients is high during metabolism. Adiponectin directly acts on the vascular endothelium and participates in energy metabolism and inflammation [53, 54]. There was a negative correlation between adiponectin and blood lipid levels. Some studies have reported that adiponectin deficiency is one of the factors leading to inflammation [55–57]. In patients with type 2 diabetes and autonomic neuropathy, complete bladder emptying does not occur, resulting in residual urine. The overgrowth of bacteria in some lesions can explain the occurrence of ASB [58]. Caregivers should protect patients from multiple comorbidities from multiple directions, carry out health education to improve patients’ diet and lifestyle, provide personalized psychological knowledge and behavioral guidance, and mobilize patients’ behavioral enthusiasm. Timely symptom evaluation and management of related diseases can delay their development, thus reducing the incidence of ASB in patients with T2DM.

## Limitations of the study

Cross-sectional studies on related topics were not included in this study, resulting in a small number of documents eventually being included; most of the people included in the studies were from developing countries. Therefore, the final meta-analysis results only represent the main risk factors for ASB in some patients with T2DM, and there may be some bias. Due to the differences in the characteristics of research objects, the number of samples, and the methods and instruments used to determine related indicators among different studies, the results of the meta-analysis are relatively heterogeneous. The exact reasons behind the reduced heterogeneity of results remain unclear. Therefore, it is suggested that more high-quality, multi-center, and large-sample original research should be conducted in the relevant direction to provide more targeted measures and generate more scientific evidence-based recommendations for patients with T2DM.

## Summary

There is no evidence that ASB treatment can reduce the incidence of UTI. Therefore, it is vital to pursue prevention as the primary strategy in patients with T2DM [58]. This study shows that age, female sex, duration of diabetes, HbA1c, hypertension, hyperlipidemia, neuropathy, and proteinuria are risk factors for ASB in type 2 diabetes; however, the source of heterogeneity of the results needs further investigation, and more relevant original research support is required in the future.

### Supplementary information

Appendix

## Abbreviations

T2DM: type 2 diabetes mellitus
ASB: asymptomatic bacteriuria
UTI: urinary tract infection
NOS: Newcastle Ottawa Scale
