# Supplementary material for: Incidence and risk factors of asymptomatic bacteriuria in patients with type 2 diabetes mellitus: a meta-analysis
Source: Endocrine. 2023 Aug 21;82(2):263–81. doi: 10.1007/s12020-023-03469-6 (PMC10543815; doi:10.1007/s12020-023-03469-6)
Supplement: Supplementary file 1 — Appendix [file 12020_2023_3469_MOESM1_ESM.docx]

**Appendix：**

1. **Search strategies:** 11 databases in Chinese and English language were searched, including:

Eleven databases, including PubMed, Web of Science, Cochrance, Embase, Ovid, Scoups, Chinese full-text journal database(CNKI), VIP database, Wan fang database, The national medical journal of China, Sinomed database. Since search strategies were the same for Chinese database, only search criteria for the representative PubMed and CNKI were listed as follows.

The search model was as follows:

**PubMed** 2023/02/03

#1 ((((((asymptomatic bacteriuria) OR (pyuria)) OR (ASB)) OR (ABU)) OR (funguria)) OR (bacteriuria)) OR (leukocyturia)

#2 ((infection) AND (asymptomatic)) AND ((((((urinary) OR (bladder)) OR (urethra)) OR (urination)) OR (urine)) OR (ureter))

#3 #1 OR #2

#4 (((((((((((Diabetes Mellitus, Type 2[Mesh]) OR ("Ketosis-Resistant Diabetes Mellitus")) OR ("Non-Insulin-Dependent Diabetes Mellitus")) OR ("Stable Diabetes Mellitus")) OR (NIDDM)) OR ("Maturity Onset Diabetes Mellitus")) OR (MODY)) OR ("Type 2 Diabetes Mellitus")) OR ("Noninsulin Dependent Diabetes Mellitus")) OR ("Maturity Onset Diabetes")) OR ("Type 2 Diabetes")) OR ("Adult-Onset Diabetes Mellitus")

#5 (((((risk factor) OR (influencing factors)) OR (Related factor)) OR (Correlation)) OR (Relevant factors)) OR (correlates)

#3 AND #4 AND #5

**CNKI** 2023/02/03

#1 (TKA =' asymptomatic bacteriuria' OR TKA=' occult bacteriuria' OR TKA=' bacteriuria' OR TKA=' pyuria' OR TKA=' leucocyturia' OR TKA=' asymptomatic urinary tract infection' OR TKA=' occult urinary tract infection' OR TKA=' asymptomatic bladder infection' OR TKA=' occult urinary tract infection' OR TKA=' asymptomatic ureteral infection' OR TKA=' occult ureteral infection')

#2 (TKA =' type 2 diabetes')

#3 (TKA =' risk factors' OR TKA =' influencing factors' OR TKA =' related factors' OR TKA =' correlation' OR TKA =' predictive factors' OR TKA =' factors' OR TKA =' related')

#1 AND #2 AND #3

**2. Conflicts of Interest**

The authors declare that they have no competing interests, and due care has been taken to ensure the integrity of the work. Neither the entire paper nor any part of its content has been published or has been accepted elsewhere. It is not being submitted to any other journal. The authors have no other relevant affiliations or financial involvement with any organization or entity with a financial interest in or financial conflict with the subject matter or materials discussed in the manuscript apart from those disclosed.

**3.Author Contributions**

All authors have made substantive contributions to this study in regard to design and implementation. Mengqiao Dai and Shan Hua: conceptualization and methodology. Mengqiao Dai: data curation and analysis and original writing. Shuqin Hu and Hu Chen: review and editing. Jiechao Yang,Dandan Geng and Weina Li: data Curation, and validation. Xiaoqin Liao is the corresponding authors for the article. All the authors read and approved the final manuscript.
